# Associations between characteristics of the patients at municipal acute bed unit admission and further transfer to hospital: a prospective observational study

**DOI:** 10.1186/s12913-020-05823-0

**Published:** 2020-10-20

**Authors:** Synnøve Karin Hernes, Valborg Baste, Kurt Arild Krokmyrdal, Silje Longva Todnem, Sabine Ruths, Ingrid Hjulstad Johansen

**Affiliations:** 1 Bergen Municipal Acute Bed Unit, Bergen, Norway; 2 National Centre for Emergency Primary Health Care, NORCE Norwegian Research Centre, Bergen, Norway; 3 Faculty of Health Studies, VID Specialized University, Bergen, Norway; 4 Voss Emergency Primary Healthcare Clinic, Voss, Norway; 5 Research Unit for General Practice, NORCE Norwegian Research Centre, Bergen, Norway; 6 Department of Global Public Health and Primary Care, University of Bergen, Bergen, Norway

**Keywords:** Community hospital, Early warning score, Intermediate care facilities, Municipal hospital, Municipal acute bed unit, Patient admission, Primary health care, Triage early warning score (TEWS)

## Abstract

**Background:**

As an alternative to acute hospitalisations, all communities in Norway are required to provide inpatient care in municipal acute bed units (MAUs) for patients who can be treated at the primary care level. Patient selection is challenging, and some patients need transfer from MAUs to hospitals. The aim of this study was to examine associations between characteristics of the patient at admission to MAU and further transfer to hospital.

**Methods:**

In a prospective observational study on all admissions to a large MAU, March 2016–August 2017, information was obtained on patient age, gender, comorbidities, drug use, reason for stay and Triage Early Warning Score (TEWS) on admission and at discharge, and length of stay. Comparison between admissions resulting in discharge to hospital, nursing home or own home were performed with chi-square and ANOVA tests. Estimated relative risks (RR) with 95% confidence interval for transfer to hospital versus being retained at primary care level was estimated for age, gender, comorbidity and TEWS in generalized linear models, crude and adjusted.

**Results:**

Two thousand seven hundred forty-four admissions were included. Mean age of the patients was 69.5 years (SD 21.9), 65.2% were women. In 646 admissions (23.6%), the patients were transferred to hospital. Male gender and TEWS > 2 were associated with transfer to hospital. Most transfers to hospital occurred within 24 h, and these patients had unchanged or increasing TEWS during their stay at MAU. When transferred to hospital 41.5% of the patients had the same reason for stay as on MAU admission, 14.9% had another reason for stay, 25.2% had a medical condition outside the treatment scope of MAU, and 18.4% needed further diagnostic clarification in hospital.

**Conclusions:**

Likelihood of transfer to hospital increased with male gender and higher TEWS on admission. Main reasons for transfer to hospital were lack of improvement and identification of clinical conditions that needed hospital care. TEWS > 2 at admission should make physicians alert to the need of close monitoring for lack of improvement.

## Background

In many countries there is an increasing burden on health services, in particular hospital services, from demographic changes and more patients living longer with chronic health problems. In Norway, municipal acute bed units (MAUs) were launched as part of the governmental Coordination Reform to reduce acute hospitalisations [1]. MAUs were established in many municipalities from 2012 and mandated by the Government from 2016. MAUs are intended for short-term stays and shall provide inpatient care for patients with clinical conditions that can be treated and cared for at the community care level, and who otherwise would have been admitted to hospital [2]. The Norwegian public health care is organised as primary health care (general practice, emergency primary health care clinics, home care, nursing homes and MAUs) and specialist health services (hospitals, outpatient specialist care, contract specialists), with mandatory gatekeeping between the service levels.

The establishment of MAUs has contributed to reduced numbers of emergency admissions to hospitals, especially for elderly patients [3]. Qualitative studies have shown that patients admitted to MAUs were more satisfied with their stay than patients admitted to hospitals [4, 5]. However, referring general practitioners (GPs) have described the selection of patients to MAU as challenging [6, 7]. Concerns have been raised about low quality of selection, particularly in emergency primary health care clinics, due to time constraints and limited patient information available [8]. Health professionals and researchers have questioned patient safety at the MAUs, partly due to risk of suboptimal diagnostics and treatment [6–10]. A hospital study in Norway showed reduced mortality among elderly patients treated in specialised geriatric hospital units compared with those treated in general medical wards [11]. For older patients, lower care level or less specialised units may lead to delayed diagnostic conclusions and reduced therapeutic quality [10].

Although patients admitted to MAUs are supposed to be treated and cared for at the community level, studies and reports show that 7.5 -15.0% of patients were transferred to hospitals [8, 12, 13], some of them with clinical conditions outside the treatment scope for MAUs [8]. To improve the selection of patients admitted to MAUs and to increase patient safety, we need to understand the reasons for hospital transfer.

## Methods

The aim of this study was to examine associations between characteristics of the patients at admission to MAU and further transfer to hospital. We conducted a prospective observational study of all admissions to the MAU in the second-largest city in Norway (278,556 inhabitants by January 2017) during the unit’s first 18 months of operation (March 2016 to August 2017). The MAU unit had 5 beds at opening, and gradually expanded to 34 beds by January 2017. The unit is co-located with the emergency primary health care clinic. Travel time by car or ambulance from MAU to the city’s two hospitals is approximately 5 min. The unit is staffed with GPs and nurses 24/7. It has its own laboratory for bedside tests, and extended test-batteries can be analysed twice daily at the nearby hospitals. X-ray of skeleton, thorax and abdomen can be taken during day and evening.

According to the national and local MAU guidelines, eligible patients must be 18 years or older and be expected to recover within 3 days. They need referral by a general practitioner or a physician at the emergency primary health care clinic. From January 2017 hospital physicians could transfer patients to the MAU within 24 h after arrival at the emergency department of the hospital.

### Data collection

During the study period, routinely registered information was extracted from the patients’ electronic medical records. For each admission, we collected administrative data about date and time of admission and discharge, workplace of referring physician, and which care level the patients were discharged to (home, nursing home or hospital).

We collected information about the patients’ age and gender, reason for stay, comorbidities and number of drugs used regularly. Reasons for stay on admission were recorded in categories corresponding to the subgroups in the mandatory reports to the Norwegian Directorate of Health: Musculoskeletal symptoms, observation, infection, dehydration, psychiatric symptoms, constipation, social causes, chronic obstructive pulmonary disease (COPD), diabetes or substance abuse.

A comorbidity was recorded if the patient had a chronic disorder included in any of the following groups: cardiovascular disease, active cancer disease, dementia or other types of reduced cognitive capacity, diabetes mellitus, obstructive lung disorder, metal illness, substance use disorder or neurological disorder. If the patient had several chronic disorders, the number of comorbidities equalled the number of groups which corresponded to at least one of the patient’s disorders.

Further, we recorded diagnoses set at discharge (ICPC-2-codes), Triage Early Warning Score (TEWS) at admission and discharge, and appropriateness of admission to MAU. TEWS was routinely measured by nurses as part of the standard clinical observation. TEWS is a validated composite triage score [14], based on judgement of the patient’s vital parameters (respiratory rate, heart rate, temperature, systolic blood pressure), level of consciousness, mobility and whether the condition is caused by a trauma. Possible sum scores range from 0 to 16. Appropriateness of care level was assessed by the responsible physician for discharge from the MAU. The physician compared reason for stay to local MAU admission guidelines, and classified the admission according to how it conformed to the guidelines: (1) Appropriate, (2) needed other care at community level, or (3) needed hospital admission.

The number of registered comorbidities was categorized as 0, 1, 2, or > 3. Length of stay was given in hours and recalculated into shorter than 1 day, 1–3 days and longer than 3 days.

Diagnoses at end of stay were coded according to ICPC-2 codes. The ICPC-2 codes were recoded into categories corresponding to reasons for stay. Some ICPC-codes represented severe conditions that were not eligible for treatment at MAU. These were recoded as “Reason for stay outside MAU’s scope”. All admissions resulting in transfer to hospital were divided into 4 subgroups: (1) Reason for stay at transfer to hospital was similar to reason for stay on admission to the MAU and within the MAU’s scope of treatment, (2) reason for stay at transfer to hospital was different from reason for stay on admission to the MAU but still within MAU’s scope of treatment, (3) reason for stay at transfer was outside MAU’s scope of treatment, and (4) the patient needed further diagnostic clarification at hospital.

### Statistical analysis

Descriptive statistics of characteristics of admissions were number, percentages, mean, standard deviation (SD), and when appropriate, median and range. Chi-squared test and ANOVA were used for comparison between admissions discharged to different care levels. Care level was dichotomized into a new variable ‘Transfer to hospital’, yes and no (returning home or being transferred to nursing home) and used as dependent variable in the statistical models. Reported TEWS ranged from 0 to 9, with a low number of cases in the range 6–9 (score 6: 31 cases, 7: 11, 8: 7, 9: 3). For the descriptive analyses TEWS was used as a continuous variable. For the other analyses TEWS 6–9 was pooled to make the analyses more robust. Generalized linear model (GLM) was applied to estimate the relative risk (RR) with 95% confidence interval (CI) for Transfer to hospital after the stay at MAU. RR was calculated for the independent variables gender (female as reference), age groups (17–52 years as reference, 53–75 years, 76–85 years and 86–102 years), comorbidities (0 as reference, 1, 2 and 3 or more comorbidities) and TEWS (0 as reference, 1, 2, 3, 4, 5, and 6 to 9) for all reasons for stay. Both crude estimates and estimates adjusted for all variables were presented. As some patients had several admissions to MAU during the study period, robust standard error estimates were used in the GLM analyses to account for dependency between admissions. Data analysis were conducted using IBM SPSS Statistics 25 and STATA (15.1).

## Results

During the study period, there were 2748 admissions to the MAU. The admissions were dispersed on 2268 individual patients. One thousand seven hundred ninety-two patients were admitted only once, 317 twice, and the remaining 159 three or more times. Four patients who received terminal palliative care, died during their stay, and were excluded from further analysis. No other patients died during the stay at the MAU.

The study thus comprised 2744 admissions, in which the patients had mean age 69.5 years (SD 21.9), median age 77 years (range 17–102), and 65.2% were women. In most admissions (*n* = 2567, 93.5%) the patient lived in the municipality the MAU was supposed to serve. In the remaining 177 admissions, the patients were tourists. At admission the most frequent reasons for stay were musculoskeletal symptoms (37.2%), observation (24.3%) and infection (19.5%). In 32.8% of the admissions, the patients had no comorbidity, and 32.2% had one comorbidity.

### Care level at discharge

After the stay at MAU, 1812 (66.0%) patients were discharged to their home, 286 (10.4%) to nursing homes, and 646 (23.6%) to hospitals (Table 1). Workplace of the referring physician had no association with where the patients were transferred to. The patients discharged to nursing homes were generally older, had more comorbidities, and used more drugs than the other patients. In the 1110 admissions where the patients were discharged from the MAU within 24 h, 434 (39.1%) were transferred to hospital. At discharge, 2507 (91.4%) of MAU admissions were considered appropriate according to the guidelines.

**Table 1:** Patient characteristics and administrative data by transfer to different care levels

	Transferred to care level										
	Total	Home			Nursing home			Hospital			***P***-value
	N	N	%		n	%		n	%		
**Total**	**2744**	**1812**	**66.0**		**286**	**10.4**		**646**	**23.6**		
**Gender**											**0.001**
Male	954	607	63.6		85	8.9		262	27.5		
Female	1790	1205	67.3		201	11.2		384	21.5		
**Number of comorbidities (*n*** **= 2712)**											**< 0.001**
0	900	671	74.5		41	4.6		188	20.9		
1	883	580	65.7		84	9.5		219	24.8		
2	609	358	58.8		85	13.9		166	27.3		
3+	320	182	56.8		70	21.9		68	21.3		
**Reason for stay (*n*** **= 2743)**											**< 0.001**
Musculoskeletal symptoms	1022	643	62.9		160	15.7		219	21.4		
Observation	667	477	71.5		53	8.0		137	20.5		
Infection	535	362	67.7		29	5.4		144	26.9		
Dehydration	128	85	66.4		10	7.8		33	25.8		
Psychiatric symptoms	93	43	46.2		7	7.6		43	46.2		
Constipation	86	62	72.1		5	5.8		19	22.1		
Social causes	83	48	57.8		17	20.5		18	21.7		
Chronic obstructive pulmonary disease	82	56	68.3		2	2.4		24	29.3		
Diabetes mellitus	33	28	84.8		0	0.0		5	15.2		
Substance abuse	14	8	57.1		2	14.3		4	28.6		
**Workplace of referring physician**											**0.312**
Emergency primary health care clinic	2464	1614	65.5		262	10.6		588	23.9		
General Practice	185	127	68.7		15	8.1		43	23.2		
Hospital	77	60	77.9		7	9.1		10	13.0		
Other	18	11	61.1		2	11.1		5	27.8		
**Length of stay**											**< 0.001**
Shorter than 1 day	1110	639	57.6		37	3.3		434	39.1		
1–3 days	1053	796	75.6		106	10.1		151	14.3		
Longer than 3 days	581	377	64.9		143	24.6		61	10.5		
**Judged appropriateness of care level**											**< 0.001**
Appropriate	2507	1747	69.7		263	10.5		497	19.8		
Needed other care at community level	82	54	65.9		22	26.8		6	7.3		
Needed hospital admission	155	11	7.1		1	0.6		143	92.3		
	**Mean**	**Mean**	**SD**	**95% CI**	**Mean**	**SD**	**95% CI**	**Mean**	**SD**	**95% CI**	
**Age**	69.5	67.0	22.6	65.9–68.0	83.1	11.7	81.7–84.4	70.6	20.9	69.0–72.2	**< 0.001**
**Number of regular medications**	5.1	4.7	4.1	4.5–4.9	6.7	3.8	6.3–7.2	5.3	4.2	5.0–5.7	**< 0.001**

### Change in reason for stay at transfer to hospital

Table 2 shows change in reason for stay between admission and transfer for the 646 patients transferred to hospital. In 268 (41.5%) transfers, the reason for stay was unchanged from the point of admission. In 96 (14.9%) transfers, the reason for stay at transfer was different from the reason for stay at admission, but still within the treatment scope of MAU. In 163 (25.2%) transfers, a severe condition beyond the scope of treatment at MAU had been discovered. For the remaining 119 (18.4%) transfers, the patient needed further diagnostic clarification in hospital. Apart from MAU admissions related to psychiatric symptoms, infections, and diabetes mellitus, at least half of the transfers to hospital were due to conditions other than those for which the patient had been admitted to MAU.

**Table 2:** Change in reason for stay between admission and transfer to hospital

Reason for stay at admission	Reason for stay at transferal to hospital								
	All hospitalised patients	Same as atMAU admission		Other reasonfor stay than atMAU admission		Diagnosesoutside MAU’sscope		Need for furtherdiagnosticclarification	
	N	n	%	n	%	n	%	n	%
Musculoskeletal symptoms	219	110	50.2	30	13.7	56	25.6	23	10.5
Infection	144	92	63.9	7	4.9	16	11.1	29	20.1
Observation	137	-^a^	–	37	27.0	58	42.3	42	30.7
Psychiatric symptoms	43	41	95.3	0	0.0	2	4.7	0	0.0
Dehydration	33	2	6.1	9	27.3	14	42.4	8	24.2
Chronic obstructive pulmonary disease	24	12	50.0	5	20.8	5	20.8	2	8.4
Constipation	19	8	42.1	0	0.0	6	31.6	5	26.3
Social causes	18	0	0.0	6	33.4	4	22.2	8	44.4
Diabetes mellitus	5	3	60.0	0	0.0	1	20.0	1	20.0
Substance abuse	4	0	0.0	2	50.0	1	25.0	1	25.0
**Total**	**646**	**268**	**41.5**	**96**	**14.9**	**163**	**25.2**	**119**	**18.4**

^a^Patients admitted to MAU for observation, but needed further diagnostic clarification at hospital, were categorized as “need for further diagnostic clarification”

### Factors associated with transfer to hospital

Patients admitted to the MAU due to psychiatric symptoms, chronic obstructive pulmonary disease, substance abuse, infection, or dehydration, were more frequently transferred to hospital than others (Table 1). Men had increased relative risk for hospitalisation (Table 3). When adjusted for age, comorbidities and TEWS, men still had 22% higher risk for hospitalisation. The relative risk for hospitalisation also increased with increasing TEWS, with a cut-off at 3 (Table 3). This effect remained rather unchanged when adjusted for gender, age, and comorbidities.

**Table 3:** Relative risk for transfer to hospital compared to home or nursing home

	Transfer to hospital crude				Transfer to hospital adjusted^**a**^	
	N	%	RR	95% CI	RR	95% CI
**Gender**						
Female	1790	21.5	1		1	
Male	954	27.5	**1.28**	**[1.11–1.48]**	**1.22**	**[1.05–1.42]**
**Age**						
17–52	621	21.1	1		1	
53–75	662	24.0	1.14	[0.91–1.42]	1.01	[0.80–1.28]
76–85	706	25.9	1.23	[0.99–1.52]	1.04	[0.82–1.32]
86–102	755	22.9	1.09	[0.87–1.35]	0.95	[0.74–1.21]
**Comorbidities**						
0	900	20.9	1		1	
1	883	24.8	1.19	[0.99–1.42]	1.15	[0.95–1.39]
2	609	27.3	**1.30**	**[1.09–1.57]**	1.15	[0.94–1.40]
3+	320	21.3	1.02	[0.80–1.29]	0.86	[0.67–1.11]
**TEWS**						
0	757	19.6	1		1	
1	632	18.7	0.95	[0.77–1.19]	0.96	[0.77–1.20]
2	589	21.7	1.11	[0.90–1.37]	1.11	[0.89–1.37]
3	341	31.1	**1.59**	**[1.28–1.98]**	**1.58**	**[1.26–1.98]**
4	154	34.4	**1.76**	**[1.35–2.30]**	**1.73**	**[1.32–2.27]**
5	63	46.0	**2.35**	**[1.74–3.19]**	**2.30**	**[1.68–3.17]**
6–9	52	53.9	**2.75**	**[2.05–3.69]**	**2.61**	**[1.90–3.60]**

^**a**^In the adjusted model *n* = 2557, due to missing in TEWS (*n* = 156) and comorbidities (additional *n* = 31)

**Fig. 1:**
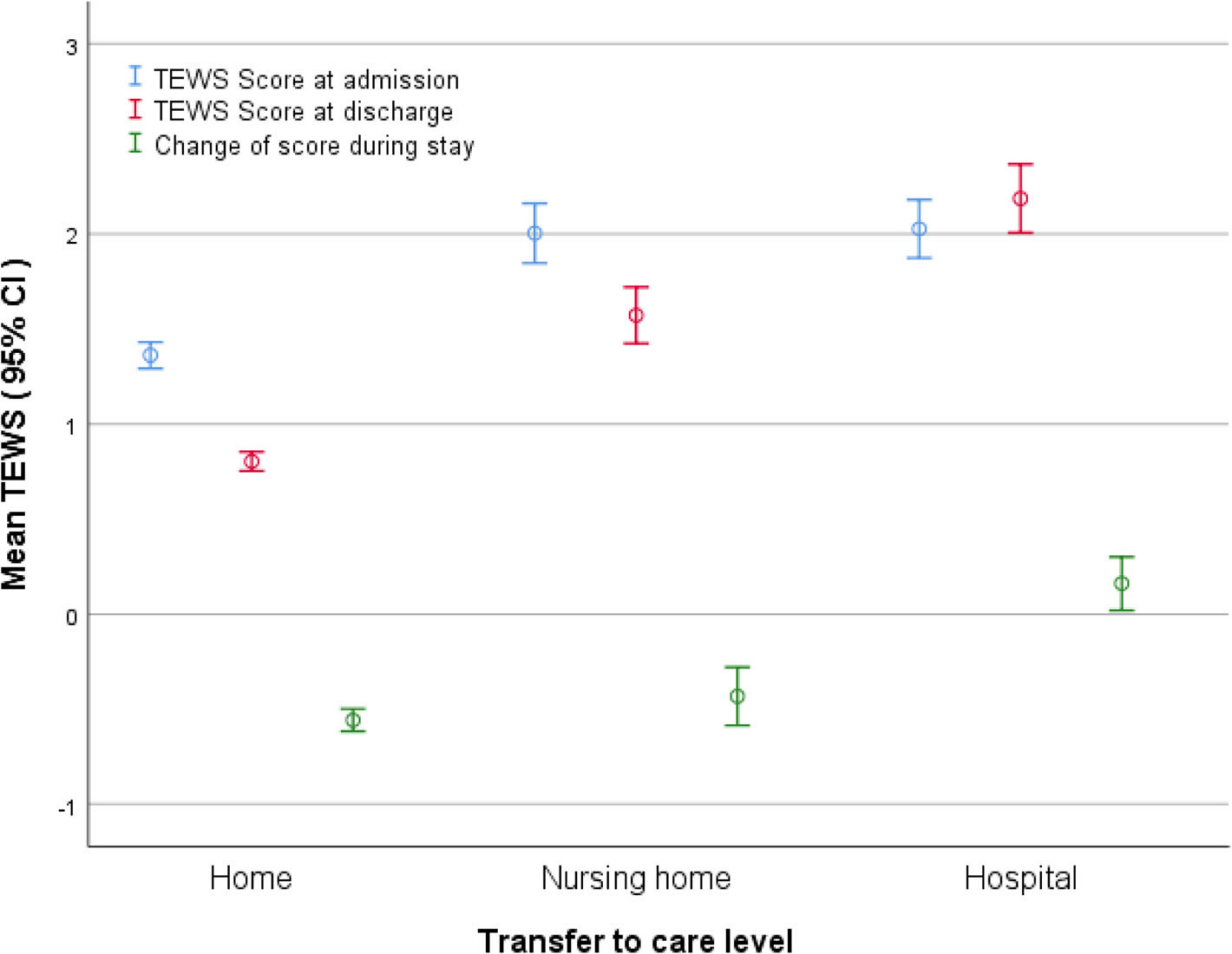
Average Triage Early Warning Score (TEWS) for patients transferred to different care levels

Development of TEWS during the stay reflected the need for hospitalisation. The patients discharged to their home had lower mean TEWS on admission (mean 1.4, range 0–9), than patients transferred to nursing homes (mean 2.0, range 0–6) and hospitals (mean 2.0, range 0–9). The patients transferred to hospital had unchanged or increased TEWS on transfer, as opposed to patients transferred to nursing home or discharged to home, whose condition improved during the stay (Fig. 1).

## Discussion

### Main findings

This prospective observational study of a Norwegian MAU showed that every fourth patient was transferred from MAU to hospital, mostly within 24 h. Patients admitted to the MAU due to psychiatric symptoms, chronic obstructive pulmonary disease, substance abuse, infection or dehydration were more frequently admitted to hospital than others. Hospitalisation was associated with male gender and TEWS > 2. Main reasons for transfer to hospital were deterioration of the clinical condition and identification of clinical conditions that needed hospital care.

### Comparison with other studies

The characteristics of the admissions in our study are mainly in accordance with patient, diagnostic and administrative characteristics of other MAUs in Norway [13]. In national statistics, more than half of the patients were admitted to MAU due to musculoskeletal symptoms, respiratory diseases and need for observation [13]. Smaller Norwegian studies have found infection as the most frequent reason for stay [8, 12]. In our study, the most frequent reason for stay was musculoskeletal symptoms. This may partly be due to co-location with the emergency primary health care clinic, which also has x-ray. Many of these patients might previously have been transferred to the orthopaedic department at a general hospital for mobilisation or, if being other places in Norway, needed admission at an orthopaedic department for further diagnostics. The patients in our study were on average younger than in other Norwegian studies [8, 12].

The observed patient transfer rate from the MAU to hospitals was 23.6%, as compared to 7.5 -15.0% reported previously [8, 12, 13]. It has been assumed that geographical distance to hospital care combined with the referring physicians’ level of experience, are important determinants for further referral [15]. Characteristics of the patient population, competence of MAU staff, distance to general hospitals, and local guidelines for admission and transfer might all contribute to the differences seen. Although it has been suggested that physicians from emergency primary health care clinics might be less adept at choosing the right patients for admission [8, 12], our study showed no association between the workplace of the referring physician and where the patient was discharged to.

In our study, most hospital transfers happened within 24 h, and there were no unexpected deaths. Rapid identification of patients in need of specialized health care might give a low risk of delayed interventions. In half of the admissions, the transfer was due to other conditions than the initial reason for stay. These conditions were frequently outside MAU’s scope for treatment. Thus, the observation time at MAU might have helped identify a more severe underlying health problem which otherwise might have gone unnoticed.

MAUs might not merely be an alternative to hospitals, but also a good alternative for patients in need of observation [6, 15, 16]. In our study, the observation at MAU uncovered a condition outside MAU’s scope for treatment in 25.2% of admissions where the patient was transferred to hospital. The corresponding proportion was 42.3% for admissions due to need for observation. Studies have shown no negative consequences of stays at MAUs as compared to stays in hospitals [16, 17]. Still, observing patients’ unclear health conditions at the MAU might increase the risk of delayed diagnosis and treatment [8, 10].

We found that patients admitted to the MAU due to psychiatric symptoms, chronic obstructive pulmonary disease, substance abuse, infection, or dehydration, were more frequently transferred to hospital than others. The effect was most pronounced for patients admitted with psychiatric symptoms, where 46.2% were transferred to hospital. More information is needed to understand this finding, and to understand the appropriate use of MAUs for patients with psychiatric symptoms. In a study of a smaller Norwegian MAU, musculoskeletal conditions were the only predictive factor for transfer to a higher care level [12]. In our study, musculoskeletal symptoms were the most common reason for transfer to nursing home. Generally, we may assume that the oldest patients need extended care, rather than advanced medical treatment. The original 3-day limit of stay at MAU was an administrative limit that has since been removed in national guideline updates [2]. According to national statistics, there has been an increase in the number of stays longer than 3 days [13]. It might be that the previous 3-day limit caused unnecessary transfers to nursing homes as well as hospitals.

In this study, male gender was associated with transfer to hospital. Higher rate of hospitalisation of men as compared to women has also been found in studies on outcomes of emergency department visits for diverticulitis [18] and renal colic [19]. The finding is puzzling, especially as the gender effect remained relatively unchanged when adjusted for age, comorbidities and TEWS. Unfortunately, our data did not allow to adjust for diagnoses. This finding should be further explored in larger studies that include clinical data.

To the best of our knowledge, no previous study on MAUs has included TEWS. Our study showed that patients transferred to nursing homes and hospitals had higher TEWS on admission than patients discharged to their home. The likelihood of transfer to hospital increased at TEWS > 2 on MAU admission. Interestingly, this sum score corresponds to the proposed TEWS’ cut-off score for significant pathology [14]. According to the TEWS’ manual patients with a sum score > 2 are considered to have significant pathology, and sum score > 4 suggests potentially life- or limb-threatening pathology. In our study the highest recorded sum score was 9, and some of the patients with highest TEWS at admission were discharged to their home or nursing homes. The patients transferred to hospitals had stable or increased TEWS during their MAU stay, as opposed to the patients discharged to their home or nursing home, whose TEWS improved. Therefore, it seems likely that physicians should be alerted when admitting patients with TEWS > 2 to MAUs. They should monitor these patients closely to capture deterioration of the clinical condition. The low sensitivity of a single triage measure to predict care level has been reported elsewhere [20, 21]. However, repeated TEWS seems to be a useful way of discovering lack of improvement.

### Strengths and limitations of the study

The strengths of this study are the large number of consecutive admissions to a MAU and the prospective gathering of complete data sets. Our results are mostly comparable to findings from national statistics. However, differences in reasons for stay and rate of transfer to hospital, especially as compared to findings in other single MAU studies [8, 12], suggest that local differences affect the reasons for stay and severity of disease among patients admitted to MAUs.

We used crude groups of chronic diseases and number of regular medications as measures for morbidity. Although presence of diseases within several diagnostic groups and use of multiple medications might be a good approximation for morbidity, the measures do not necessarily reflect frailty or severity of disease. Another limitation of the study is that although reason for stay seemed to affect transfer rate to hospital, the generalized linear model did not allow simultaneous analysis for TEWS and reason for stay, due to many and small subcategories. We therefore performed a logistic regression that allowed for the inclusion of both variables, in addition to age, gender and comorbidities. The results of the regression were similar to the results from the statistic model, and we therefore chose not to include the results from the logistic regression in the manuscript.

## Conclusions

Male gender and TEWS > 2 were associated with further transfer to hospital. Main reasons for transfer to hospitals were lack of improvement and identification of other health conditions that needed hospital treatment. This suggests that physicians at MAUs should be alerted and monitor closely patients who have TEWS > 2 when admitted. The gender difference found in this study should be further explored in other studies.

## Data Availability

The original datasets generated and analysed during the current study are not publicly available due to restrictions in the permission from REC North. Aggregated datasets are available from the corresponding author on reasonable request.

## Abbreviations

MAU: Municipal acute bed unit
GP: General practitioner
TEWS: Triage Early Warning Score
